# Supplementary material for: Diverse fates of uracilated HIV-1 DNA during infection of myeloid lineage cells
Source: eLife. 2016 Sep 20;5:e18447. doi: 10.7554/eLife.18447 (PMC5030084; doi:10.7554/eLife.18447)
Supplement: Supplementary file 1. — Infected MDMs containing proviruses were sorted into two populations according to GFP fluorescence at 14 days post infection and then activated with the indicated cytokines. Mutation frequencies are calculated based on total nucleotides sequenced (i.e. frequency = mutated nucleotide count/total nucleotides). RT-PCR was used to amplify extracellular viral RNAs into cDNA prior to clonal sequencing. DOI: http://dx.doi.org/10.7554/eLife.18447.023 [file elife-18447-supp1.docx]

| Sorted  population | Region | Cytokine | Clones  sequenced | Base pairs sequenced | Total point mutations | Transitions mutations | Transversion mutations | I+D |
| --- | --- | --- | --- | --- | --- | --- | --- | --- |
| GFP^-^ | LTR | None^d^ | 5 | 1,540 | 0 | 0 | 0 | 0 |
|  |  | IFNγ | 5 | 1,540 | 0 | 0 | 0 | 0 |
|  |  | IL-4 | 14 | 4,312 | 0 | 0 | 0 | 0 |
|  |  |  |  |  |  |  |  |  |
|  | *env* | None^c^ | 10 | 4,080 | 48 | 7 | 0 | 3 |
|  |  | IFNγ | 6 | 2,448 | 56 | 9 | 3 | 7 |
|  |  | IL-4 | 8 | 3,264 | 2 | 0 | 0 | 0 |
|  |  |  |  |  |  |  |  |  |
| GFP^+^ | LTR | None | 7 | 2,156 | 0 | 0 | 0 | 0 |
|  |  | IFNγ | 7 | 2,156 | 0 | 0 | 0 | 0 |
|  |  | IL-4 | 7 | 2,156 | 0 | 0 | 0 | 0 |
|  |  |  |  |  |  |  |  |  |
|  | *env* | None | 11 | 4,488 | 0 | 0 | 0 | 0 |
|  |  | IFNγ | 9 | 3,672 | 0 | 0 | 0 | 0 |
|  |  | IL-4 | 9 | 3,672 | 0 | 0 | 0 | 0 |
