# Supplementary material for: Diverse fates of uracilated HIV-1 DNA during infection of myeloid lineage cells
Source: eLife. 2016 Sep 20;5:e18447. doi: 10.7554/eLife.18447 (PMC5030084; doi:10.7554/eLife.18447)
Supplement: Supplementary file 3. — DOI: http://dx.doi.org/10.7554/eLife.18447.025 [file elife-18447-supp3.docx]

| GFP forward | CGACTTCTTCAAGTCCGCCA |
| --- | --- |
| GFP reverse (79bp) | CTTGTAGTTGCCGTCGTCCT |
| GFP reverse (281bp) | TGCCGTCCTCGATGTTGTG |
| GFP reverse (363bp) | CTCAGGTAGTGGTTGTCGGG |
| GFP reverse (471bp) | TTGTACAGCTCGTCCATGCC |
| GFP probe | FAM-CCCGAAGGCTACGTCCAGGAGCGC-BHQ2 |
| Early forward | GCTAACTAGGGAACCCACTGCTT |
| Early reverse | CAACAGACGGGCACACACTGCTT |
| Early probe | FAM-AGCCTCAATAAAGCTTGCCTTGAGTGCTTC-BHQ2 |
| Alu forward | GCCTCCCAAAGTGCTGGGATTACAG |
| gag forward (6F) | CATGTTTTCAGCATTATCAGAAGGA |
| gag reverse (84R) | TGCTTGATGTCCCCCCACT |
| gag probe | FAM-CCACCCCACAAGATTTAAACACCATGCTAA-BHQ2 |
| LRT forward (MH531) | TGTGTGCCCGTCTGTTGTGT |
| LRT reverse (MH532) | GAGTCCTGCGTCGAGAGATC |
| LRT probe | FAM-CAGTGGCGCCCGAACAGGGA-BHQ2 |
| 2LTR forward (MH535) | AACTAGGGAACCCACTGCTTAAG |
| 2LTR reverse (MH536) | TCCACAGATCAAGGATATCTTGTC |
| 2LTR probe (MH603) | FAM-ACACTACTTGAAGCACTCAAGGCAAGCTTT-BHQ2 |
| pol forward (mf299) | GCACTTTAAATTTTCCCATTAGTCCTA |
| pol reverse (mf302) | CAAATTTCTACTAATGCTTTTATTTTTTC |
| pol probe (mf348) | FAM-AAGCCAGGAATGGATGGCC-MGBNFQ |
| RPP30 forward | GATTTGGACCTGCGAGCG |
| RPP30 reverse | GCGGCTGTCTCCACAAGT |
| RPP30 probe | VIC-CTGACCTGAAGGCTCT-MGBNFQ |
| vpr sense | GGAAACTGACAGAGGACAGGTGGAACAAGCCCC |
| 5LTRout | CACACAAGGCTAYTTCCCTGA |
| Mod VQA R | TTTTTTGAGGCTTAAGCAGTGGGTTCCCTA |
| ES7 | CTGTTAAATGGCAGTCTAGC |
| ES8 | CACTTCTCCAATTGTCCCTCA |
| Nesty8 | CATACATTGCTTTTCCTACT |
| DLoop | GTCTAGCAGAAGAAGAGG |
| vpr antisense | GGGGCTTGTTCCACCTGTCCTCTGTCAGTTTCC |
| vif sense | GGAAACTGACAGAGGACAGGTGGAACAAGCCCC |
| vpr antisense | GGGGCTTGTTCCACCTGTCCTCTGTCAGTTTCC |
