# Supplementary material for: Diverse fates of uracilated HIV-1 DNA during infection of myeloid lineage cells
Source: eLife. 2016 Sep 20;5:e18447. doi: 10.7554/eLife.18447 (PMC5030084; doi:10.7554/eLife.18447)
Supplement: Supplementary file 4. — Abbreviations: M; Male, F; Female, ABC; abacavir, DTG; dolutegravir, 3TC; lamivudine, DRV; darunavir, FTC; emtricitabine, r; ritonavir, ATV; atazanavir, TDF; tenofovir disoproxil fumarate, EVG/c; elvitegravir/cobicistat, EFV; efavirenz, N/A; not applicable. DOI: http://dx.doi.org/10.7554/eLife.18447.026 [file elife-18447-supp4.docx]

| Participant ID | Age | Sex | Time since diagnosis (months) | Time on ART (months) | CD4 count (cells/μL) | Viral load (copies/mL) | ART Regimen |
| --- | --- | --- | --- | --- | --- | --- | --- |
| 1 | 53 | M | 132 | 132 | 573 | <20 | ABC/3TC/DTG |
| 2 | 55 | M | 156 | 112 | 269 | <20 | FTC DTG DRV/r |
| 3 | 55 | M | 336 | 264 | 586 | <20 | FTC/TDF DTG ATV/r |
| 4 | 43 | F | 156 | 134 | 598 | <20 | FTC/TDF/EVG/c |
| 5 | 43 | M | 132 | 124 | 838 | <20 | ABC/3TC/EFV |
| 6 | 41 | M | 279 | 264 | 489 | <20 | ABC/3TC/DTG |
| 1Bpre | 35 | M | N/A | N/A | 406 | 29,245 | None |
| 1Bpost |  |  | 10 | 10 | 413 | 88 | FTC/TDF/EFV |
